# Quantification of Respiratory and Muscular Perceived Exertions as Perceived Measures of Internal Loads During Domestic and Overseas Training Camps in Elite Futsal Players

**DOI:** 10.3389/fpsyg.2021.751030

**Published:** 2022-01-28

**Authors:** Yu-Xian Lu, Filipe M. Clemente, Pedro Bezerra, Zachary J. Crowley-McHattan, Shih-Chung Cheng, Chia-Hua Chien, Cheng-Deng Kuo, Yung-Sheng Chen

**Affiliations:** ^1^Graduate Institute of Athletics and Coaching Science, National Taiwan Sport University, Taoyuan, Taiwan; ^2^Department of Exercise and Health Sciences, University of Taipei, Taipei City, Taiwan; ^3^Escola Superior de Desporto e Lazer, Instituto Politécnico de Viana do Castelo, Rua Escola Industrial e Comercial de Nun’Álvares, Viana do Castelo, Portugal; ^4^Instituto de Telecomunicações, Delegação da Covilhã, Lisbon, Portugal; ^5^Research Center in Sports Performance, Recreation, Innovation and Technology (SPRINT), Melgaço, Portugal; ^6^Discipline of Sport and Exercise Science, Faculty of Health, Southern Cross University, Lismore, NSW, Australia; ^7^Exercise and Health Promotion Association, New Taipei City, Taiwan; ^8^Department of Medical Research, Taipei Veterans General Hospital, Taipei City, Taiwan; ^9^Department of Medicine, Taian Hospital, Taipei City, Taiwan; ^10^Tanyu Research Laboratory, Taipei City, Taiwan

**Keywords:** monitoring training loads, training camps, futsal training, Borg CR10, heart rate, total distance covered

## Abstract

**Background:**

The rating of perceived exertion (RPE) scales with respiratory and muscular illustrations are recognized as simple and practical methods to understand individual psychometric characteristics in breathing and muscle exertion during exercise. However, the implementation of respiratory and muscular RPE to quantify training load in futsal training camps has not been examined. This study investigates respiratory and muscular RPE relationships during domestic training camps (DTC) and overseas training camps (OTC) in an under 20 futsal national team.

**Methods:**

Data collected from eleven field players were used for comparison in this study (age = 18.7 ± 0.7 years, height = 171.9 ± 6.1 cm, body weight = 64.2 ± 8.4 kg). All players reported Borg CR10 RPE (RPE_CR10_) and 7-scales respiratory RPE (RPE_respiration_) and muscular RPE (RPE_muscle_) (Dalhousie pictorial scales) after training sessions and matches. Additionally, total distance covered (TD) and training impulse (TRIMP) were used to quantify external and internal loads *via* the Polar Team Pro system. Paired-sample *t*-tests were used to compare the RPE_CR10_, RPE_respiration_, RPE_muscle_, TD, and TRIMP between DTC and OTC. Furthermore, linear regression was performed to determine the relationships among all RPE scales, TD, and TRIMP.

**Results:**

The RPE_CR10_ (*p* = 0.047), TD (*p* < 0.001), and TRIMP (*p* < 0.001) showed significant difference between DTC and OTC. Furthermore, linear regression analyses showed significant correlation between RPE_respiration_ and RPE_muscle_ (DTC: *r* = 0.857, *p* = 0.006; OTC: *r* = 0.924, *p* < 0.001), RPE_CR10_ and RPE_respiration_ (DTC: *r* = 0.857, *p* = 0.001; OTC: *r* = 0.863, *p* = 0.001), and RPE_CR10_ and RPE_muscle_ (DTC: *r* = 0.906, *p* < 0.001; OTC: *r* = 0.900, *p* < 0.001).

**Conclusion:**

Pictorial RPE_respiration_ or RPE_muscle_ can be used as an alternative to quantify perceived measures of internal loads during DTC and OTC in futsal players. Interpretation of perceived measures of training load and cardiac-related responses in association with external training loads during short-term training camps would be useful in further understanding the demands of futsal players’ experience in these circumstances.

## Introduction

The monitoring of training loads is a practical method to evaluate physical adaptation and recovery status during a training period (Bourdon et al., 2017). Methodologies to monitor training loads can be divided into internal and external measures (Impellizzeri et al., 2019). Internal measures of load can be evaluated *via* self-report measures [i.e., rating of perceived exertion (RPE)] or objective measures of physiological response [i.e., heart rate (HR) and blood lactate concentration] (Clemente et al., 2019). Conversely, external load measures that assist in measuring locomotion profiles *via* microelectromechanical systems (e.g., global navigation satellite system, inertial measurement unit, or local positioning system) can be used to evaluate physical impact and physiological strains during training sessions and competitions (Halson, 2014).

Futsal is a high-intensity intermittent and strenuous indoor sports (Barbero-Alvarez et al., 2008) requiring both aerobic and anaerobic energy systems to maintain vigorous physical and mental conditions (Naser et al., 2017). Futsal competitions are incredibly high in intensity and often cause intensive physiological and psychological strains to the players. It has been reported that high-speed running (18.1–25 km.h^–1^) and sprinting (>25 km.h^–1^) account for 22.6% of the total distance covered (TD) during a competitive futsal match. Additionally, field players have average HR responses between 170 and 190 beats.min^–1^ in Liga Nacional de Futbol Sala (around 83% of maximal HR during the match time), representing a high cardiovascular load on players (Barbero-Alvarez et al., 2008). This cardiovascular load results from rapid changes in various activities every 8–9 s (e.g., high-speed running, sprints, change of direction, and lateral and backward activity) during matches (Álvarez et al., 2009).

Much like during match scenarios, monitoring the demands of futsal training is also important to understand the accumulation of the training load imposed on an individual player (Clemente et al., 2020). Among the possible methods of monitoring internal load, the RPE is a simple and practical tool to quantify since it is a valid, low-cost, and reliable method in measuring exercise intensity (Haddad et al., 2017). The RPE is based on psychological perception in response to training and/or exercise stimuli. An 11-points scale version from 0 (no exertion) to 10 (maximal effort), Borg CR10 scale (RPE_CR10_), is developed to evaluate physiological and psychological perceptions in response to exercise strain in laboratory and field settings (Foster et al., 2001). Subsequently, the RPE_CR10_ in respect to the exercise or training duration was used to quantify internal training load in sports training (Foster et al., 2001). This method has been extensively used in team sports, such as soccer (Impellizzeri et al., 2004; Clemente et al., 2019) and futsal players (Chen et al., 2020; Clemente et al., 2020; Stochi de Oliveira and Borin, 2021) due to convenience and user-friendliness.

The respiratory (RPE_respiration_) and muscular (RPE_muscle_) perceived exertions are variants of RPE scales. The RPE_respiration_ and RPE_muscle_ primarily focus on the breathing efforts and leg fatigue during exercise, respectively (Pianosi et al., 2014, 2015). The benefit of using both the RPE_respiration_ and RPE_muscle_ scales is that they identify specific characteristics of psychophysiological responses in cardiopulmonary and leg muscle performance. The advantage of using different RPE scales to quantify training load is related to the sensitivity of the measurement. In terms of Dalhousie pictorial scales, seven different illustrations for dyspnea (chest tightness, throat closure, breathing effort, etc.) and perceived leg exertion (light leg, heavy leg, soft leg, etc.) are used to represent different physiological strains in responses to different exercise loads. The validity to use Dalhousie scales to rate respiratory and leg muscle exertions during exercise activity has been reported in pediatric populations, compared to RPE_CR10_ measure.

In terms of specific training sessions, physical demands may activate different sensory feedbacks and physiological constraints in target body systems during the performance. McLaren et al. (2016) previously reported that the sensitivity of RPE_respiration_ was different from RPE_muscle_ during incremental cycling and treadmill running in university soccer players. Furthermore, Wright et al. (2020) recently demonstrated that young female soccer players experienced stronger perceived exertions in breathing and muscular engagement during fitness and resistance training, respectively. However, no difference in respiratory and muscular perception during soccer training sessions and matches has been observed. Wright et al.’s (2020) study implied the different perceptions of sensory sources to specific training types in this population. However, the application of these measures in futsal training lacks sufficient investigation and requires further elucidation.

Futsal training camps include team preparation, player selection, building and developing team tactics and formations, physical and mental preparation, and team squad readiness. In general, domestic training camps (DTC) provide benefits for testing squad members in individual and team performance, periodizing physical and mental preparation, and tactical strategies prior to tournaments (Clemente et al., 2020). Conversely, overseas training camps (OTC) have advantages in creating a simulative environment of official tournaments and thus familiarizing players with the usual intensity of psychophysiological responses during competitions (Lu et al., 2019; Chen et al., 2020). However, a comparative study to examine training loads between DTC and OTC has not been reported elsewhere.

In light of the above, this study compares the different training measures during DTC and OTC in under 20 (U-20) male futsal players, and examines relationships between the RPEs and traing impulse (TRIMP)/TD. It was hypothesized that there would be significant differences in measured variables between DTC and OTC. The secondary hypothesis was that the relationship between perceived exertions and TRIMP/TD would be established during DTC and OTC.

## Materials and Methods

### Participants

Eleven male futsal field players from a national U-20 futsal team voluntarily participated in this study (age = 18.7 ± 0.7 years, height = 171.9 ± 6.1 cm, body weight = 64.2 ± 8.4 kg). All players signed informed consent forms and were familiarized with the procedures in reporting RPE values. This study was approved by the Human Ethics Committee of the University of Taipei (UT-IRB-2018-068) and undertaken in accordance with the Declaration of Helsinki and its later amendment.

### Design and Procedure

**FIGURE 1:**
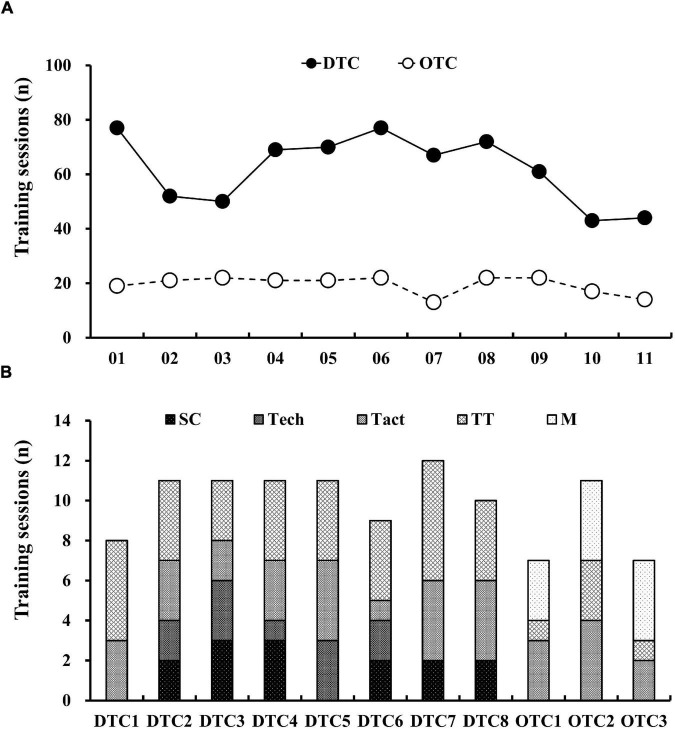
The total number of training sessions during domestic and overseas training camps. **(A)** The total number of training sessions for each individual player. **(B)** The total number of training sessions for the group. DTC, domestic training camps; OTC, overseas training camps; S&C, strength and conditioning sessions; Tech, technical training sessions; Tact, tactical training sessions; TT, technical and tactical training sessions; M, friendly match.

This was a prospective cohort study to observe the training loads during futsal training camps. The training camps consisted of eight short-term DTC (including 83 training sessions, total training duration = 149.23 h) and three short-term OTC (including 14 training sessions and 11 friendly matches, total training/match duration = 41.87 h). The description of the study period and the individual exposure time to training are presented in Tables 1, 2, respectively. Figure 1 illustrates the number and types of training sessions during each training camp. The training loads during the training camps were assessed *via* (1) perceived internal load = RPE_CR10_, RPE_respiration_, RPE_muscle_; (2) HR-related internal load = TRIMP; and (3) external load = TD. During all training sessions and friendly matches, all players wore microsensors HR monitors on the chest (Polar Team Pro, Polar Electro, Kemple, Finland). The microsensor and chest strap were marked with a unique jersey number throughout the training camps. HR responses and activity profiles were recorded and used to calculate TRIMP and TD for subsequent data processes. For the RPE scales, players reported individual perception of RPE_CR10_, RPE_respiration_, and RPE_muscle_ responses within 30 min after training sessions or friendly matches (Impellizzeri et al., 2004). The team sports trainer asked the players the following three questions: (1) How hard was your training session? (2) How does your breathing feel? (3) How do your legs feel? Subsequently, session RPE_CR10_ (sRPE_CR10_), session RPE_respiration_ (sRPE_respiration_), and session RPE_muscle_ (sRPE_muscle_) were used to calculate training loads. The sRPE was calculated based on the RPE scales × time of training sessions or matches (Foster et al., 2001).

**TABLE 1:** The description of study period.

Training camps	Duration (days)	Interval between the training camp (days)
DTC1	7	–
DTC2	7	44
DTC3	7	14
DTC4	7	21
DTC5	7	12
OTC1	4	8
DTC6	5	83
DTC7	6	10
DTC8	5	16
OTC2	6	9
OTC3	9	43

*DTC, domestic training camps; OTC, overseas training camps.*

**TABLE 2:** The individual exposure time to training during the training camps.

Player	DTC1 (min)	DTC2 (min)	DTC3 (min)	DTC4 (min)	DTC5 (min)	DTC6 (min)	DTC7 (min)	DTC8 (min)	OTC1 (min)	OTC2 (min)	OTC3 (min)
01	344	1291	1204	1189	1162	951	1208	917	686	623	793
02	361	1051	1204	1189	1262	–	–	783	686	834	793
03	115	1151	–	–	1142	699	1208	783	686	856	875
04	585	1291	1204	1189	1262	262	972	783	686	749	875
05	585	1163	763	1189	1042	951	1075	783	686	856	766
06	699	1291	1204	1189	927	951	1208	783	686	844	875
07	596	1169	1204	1080	1262	541	780	783	686	702	–
08	820	1169	242	1189	1262	951	1208	783	686	834	875
09	820	933	1204	1189	689	951	1030	–	686	856	875
10	465	671	894	1080	1262	–	–	475	686	586	556
11	–	–	1204	977	–	541	1006	783	–	706	875

*DTC, domestic training camps; OTC, overseas training camps.*

### Rating of Perceived Exertion

Each individual’s RPE responses were recorded using the Borg CR10 scale and Dalhousie pictorial scale to quantify RPE_CR10_, RPE_respiration_, and RPE_muscle_, respectively. The RPE_CR10_ is a brief version of 0–10 points scale, modified from the original Borg RPE (Borg, 1998). The RPE_CR10_ is a visual analog scale that is numerically represented as 0 being “not at all” and 10 being “extremely intense.” Additionally, the Dalhousie pictorial scales consisted of seven cartoon pictures to reflect perceived exertion in breathing effort and leg sensation during exercise. The lowest value of 1 represented “very light feeling” and the highest value of 7 represented “extremely intense feeling” (Pianosi et al., 2014, 2015, 2016). In our study, the 7-points Dalhousie pictorial scale was used to assess the respiratory and muscular RPE as it is correlated with respiratory and muscular exertions during exercise in adolescents (median value of Spearman’s *r* > 0.9) (Pianosi et al., 2015). Subsequently, all RPE scores were multiplied by the time of training sessions and friendly matches as session RPE values (Foster et al., 2001). The participants were informed of the definition of RPE scales on the first registration day and were afforded the opportunity to practice reporting RPE values during the first DTC.

### Training Impulse

Polar microsensors recorded the exercising HR responses during training and matches and were used to calculate TRIMP. The Edward’s method equation was used (Edwards, 1993) following the formula:


(1) TRIMP=Time⁢in⁢HR⁢zone⁢ 1*1+Time⁢in⁢HR⁢zone⁢ 2*2

+T⁢i⁢m⁢e⁢i⁢n⁢H⁢R⁢z⁢o⁢n⁢e⁢ 3*3+T⁢i⁢m⁢e⁢i⁢n⁢H⁢R⁢z⁢o⁢n⁢e⁢ 4*4

+T⁢i⁢m⁢e⁢i⁢n⁢H⁢R⁢z⁢o⁢n⁢e⁢ 5*5

The HR zones were defined as 50–59% of maximal HR (HR_max_), 60–69% of HR_max_, 70–79% of HR_max_, 80–89% of HR_max_, and 90–100% of HR_max_ as HR zone 1, HR zone 2, HR zone 3, HR zone 4, and HR zone 5, respectively. The HR responses below 50% of HR_max_ were excluded in data collection. The HR_max_ was determined using individual peak HR responses during the Yo-Yo intermittent recovery level 1 test in the first DTC.

### Total Distance

The TD is a sum of the traveled distance during training sessions and friendly matches. The same Polar Team Pro system (Polar Electro, Kemple, Finland) that is used for HR monitoring was used to record each player’s traveled distance. Each player was mounted with a microsensor containing a 3-dimensional accelerometer, a gyroscope, and a digital compass that sampled at a rate of 200 Hz.

### Statistical Analyses

The descriptive data were presented as means ± standard deviations (SD). Furthermore, the coefficient of variation (CV) was calculated for group or individual variability across the training camps. The average values of means and CV during a single training camp were used for statistical analyses. The normality of study variables was examined with the Kolmogorov–Smirnov test. Paired *t*-tests were used to compare the group average value of measured variables between DTC and OTC (data points: DTC = 682 data vs. OTC = 214 data). Standard differences of variables were examined by using Cohen’s *d* effect size (ES). The standardized differences of the ES were interpreted as trivial (0.0–0.2), small (0.2–0.6), moderate (0.6–1.2), large (1.2–2.0), or very large (>2.0) (Hopkins et al., 2009). Linear regression analysis was used to examine the relationship among (1) sRPE_CR10_, sRPE_respiration_, and sRPE_muscle_; and (2) between perceived exertions (sRPE_CR10_, sRPE_respiration_, and sRPE_muscle_) and TRIMP/TD training loads. Significant differences between the means were set as *p* < 0.05. All statistical analyses were performed by SPSS version 25.0 software for Windows (IBM Corp, Armonk, NY, United States).

## Results

### Comparisons Between Domestic and Overseas Training Camps

**FIGURE 2:**
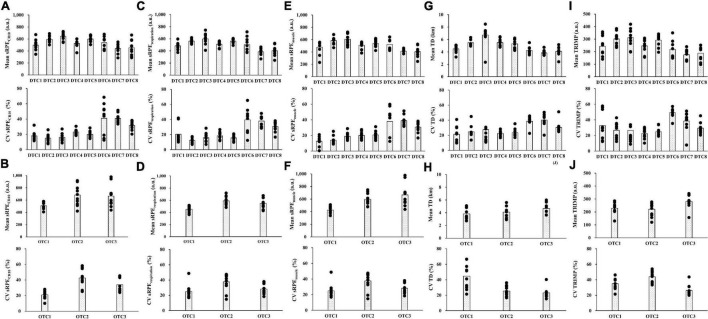
The individual and group values of measured variables across the study period. Scatterplots present individual values during each training camp whereas bar charts present group average during each training camp. **(A)** Mean and CV sRPE_CR10_ during the DTC; **(B)** mean and CV sRPE_CR10_ during the OTC; **(C)** mean and CV sRPE_respiration_ during the DTC; **(D)** mean and CV sRPE_respiration_ during the OTC; **(E)** mean and CV sRPE_muscle_ during the DTC; **(F)** mean and CV sRPE_muscle_ during the OTC; **(G)** mean and CV total distance covered during the DTC; **(H)** mean and CV total distance covered during the OTC; **(I)** mean and CV training impulse during the DTC; and **(J)** mean and CV training impulse during the OTC. sRPE_CR10_, session rating of perceived exertion CR10; sRPE_respiration_, session respiratory perceived exertion; sRPE_muscle_, session muscular perceived exertion; TD, total distance covered; TRIMP, training impulse; DTC, domestic training camps; OTC, overseas training camps. CV, coefficient of variation.

Individual and group values of TD, TRIMP, sRPE_CR10_, sRPE_respiration_, and sRPE_muscle_ during DTC and OTC training camps are presented in Figure 2.

For the pairwise comparisons between DTC and OTC, the result showed that TD [*t* = 13.592, *p* < 0.001, ES = 2.79 (very large)] and TRIMP [*t* = 7.357, *p* < 0.001, ES = 1.12 (moderate)] during DTC were significantly higher than that of OTC. All RPE scales demonstrated higher absolute values. However, only RPE_CR10_ [*t* = −2.260, *p* = 0.047, ES = −0.80 (moderate)] and sRPE_CR10_ [*t* = 2.352, *p* = 0.041, ES = −0.88 (moderate)] had significant differences (Table 3).

**TABLE 3:** The perceived exertion of CR10, respiratory and muscular scales, training impulse, and total covering distance during the domestic and overseas training camps.

	Domestic camps		Overseas camps				Effect size (90% CI)	QI for effect magnitude (mean difference; ±90% CI)
	Mean ± SD	CV (%)	Mean ± SD	CV (%)	Difference	*p*-Value		
RPE_CR10_ (a.u.)	4.92 ± 0.55	11.23	5.56 ± 0.94	16.97	–0.64	0.047	−0.80 (−1.55 – −0.09); moderate	Very likely (1.2/3.3/95.5)
RPE_respiration_ (a.u.)	4.58 ± 0.36	7.90	4.79 ± 0.48	10.08	–0.21	0.183	−0.48 (−1.20 – 0.22); small	Likely (4.2/14.0/81.8)
RPE_muscle_ (a.u.)	4.67 ± 0.30	6.4	5.05 ± 0.78	15.53	–0.39	0.156	−0.62 (−1.36 – 0.09); moderate	Likely (5.3/5.9/88.8)
sRPE_CR10_ (a.u.)	532.73 ± 60.81	11.42	615.01 ± 112.63	18.31	–82.27	0.041	−0.88 (−1.64 – −0.16); moderate	Very likely (1.1/2.5/96.3)
sRPE_respiration_ (a.u.)	494.83 ± 44.33	8.96	528.37 ± 56.53	10.70	–33.54	0.115	−0.64 (−1.37 – 0.07); moderate	Likely (2.7/8.9/88.4)
sRPE_muscle_ (a.u.)	504.37 ± 40.84	8.10	559.76 ± 91.05	16.27	–55.38	0.094	−0.76 (−1.51 – −0.05); moderate	Likely (3.0/4.3/92.7)
TRIMP (a.u.)	410.21 ± 82.71	20.16	319.64 ± 73.33	22.94	90.56	<0.001	1.12 (0.38 – 1.91); large	Most likely (99.8/0.2/0)
TD (km)	8.44 ± 0.79	9.35	5.61 ± 1.13	20.07	2.833	<0.001	2.79 (1.85 – 3.91); very large	Most likely (99.9/0/0)

*RPE_CR10_, rating of perceived exertion CR10; RPE_respiration_, respiratory perceived exertion; RPE_muscle_, muscular perceived exertion; sRPE_CR10_, session RPE_CR10_; sRPE_respiration_, session RPE_respiration_; sRPE_muscle_, session RPE_muscle_; SD, standard deviation; QI, qualitative inferences; CV, coefficient of variation; CI, confidence interval; TRIMP, training impulse; TD, total distance covered.*

### Linear Regressions of Rating of Perceived Exertions, Total Covering Distance, and Training Impulse

**FIGURE 3:**
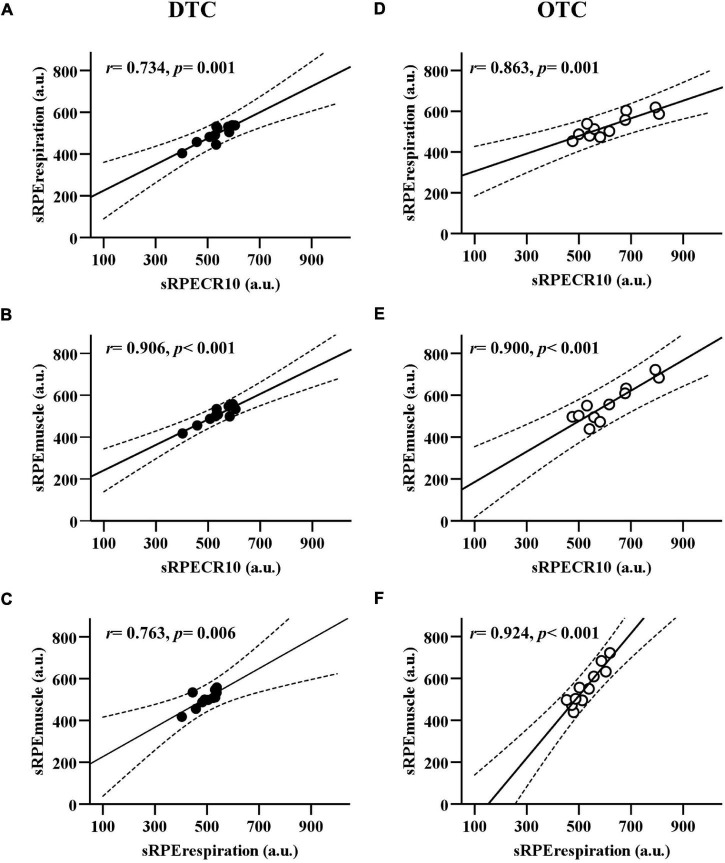
The linear regression between sRPE_CR10_, sRPE_respiration_, and sRPE_muscle_ during domestic and overseas training camps. **(A)** Comparison between sRPE_CR10_ and sRPE_respiration_ during DTC. **(B)** Comparison between sRPE_CR10_ and sRPE_muscle_ during DTC. **(C)** Comparison between sRPE_respiration_ and sRPE_muscle_ during DTC. **(D)** Comparison between sRPE_CR10_ and sRPE_respiration_ during OTC. **(E)** Comparison between sRPE_CR10_ and sRPE_muscle_ during OTC. **(F)** Comparison between sRPE_respiration_ and sRPE_muscle_ during OTC. sRPE_CR10_, session rating of perceived exertion CR10; sRPE_respiration_, session respiratory perceived exertion; sRPE_muscle_, session muscular perceived exertion; DTC, domestic training camps; OTC, overseas training camps.

Figure 3 presents the linear regression among the sRPE_CR10_, sRPE_respiration_, and sRPE_muscle_. The sRPE_CR10_ demonstrated a good positive association to sRPE_respiration_ during DTC (*r* = 0.857, *p* = 0.001) and OTC (*r* = 0.863, *p* = 0.001). The sRPE_CR10_ demonstrated excellent positive association to sRPE_muscle_ during DTC (*r* = 0.906, *p* < 0.001) and OTC (*r* = 0.900, *p* < 0.001). Additionally, the sRPE_respiration_ demonstrated good positive association to sRPE_muscle_ during DTC (*r* = 0.763, *p* = 0.006) and OTC (*r* = 0.924, *p* < 0.001).

**FIGURE 4:**
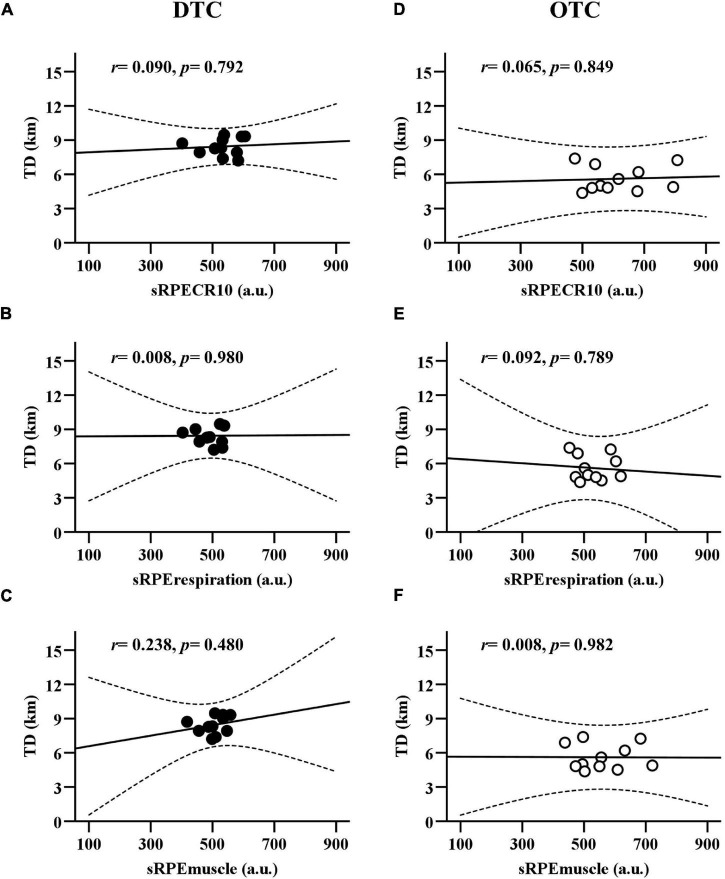
The linear regression between the RPE (RPE_CR10_, RPE_respiration_, and RPE_muscle_) and the total distance covered during domestic and overseas training camps. **(A)** Comparison between TD and sRPE_CR10_ during DTC. **(B)** Comparison between TD and sRPE_respiration_ during DTC. **(C)** Comparison between TD and sRPE_muscle_ during DTC. **(D)** Comparison between TD and sRPE_CR10_ during OTC. **(E)** Comparison between TD and sRPE_respiration_ during OTC. **(F)** Comparison between TD and sRPE_muscle_ during OTC. TD, total distance covered; sRPE_CR10_, session rating of perceived exertion CR10; RPE_respiration_, session respiratory perceived exertion; RPE_muscle_, session muscular perceived exertion; DTC, domestic training camps; OTC, overseas training camps.

In Figure 4, the sRPE_CR10_, sRPE_respiration_, and sRPE_muscle_ demonstrated poor positive association to TD during DTC (sRPE_CR10_: *r* = 0.090, *p* = 0.792; sRPE_respiration_: *r* = 0.008, *p* = 0.980; sRPE_muscle_: *r* = 0.238, *p* = 0.480) and OTC (sRPE_CR10_: *r* = 0.065, *p* = 0.849; sRPE_respiration_: *r* = 0.092, *p* = 0.789; sRPE_muscle_: *r* = 0.008, *p* = 0.982).

**FIGURE 5:**
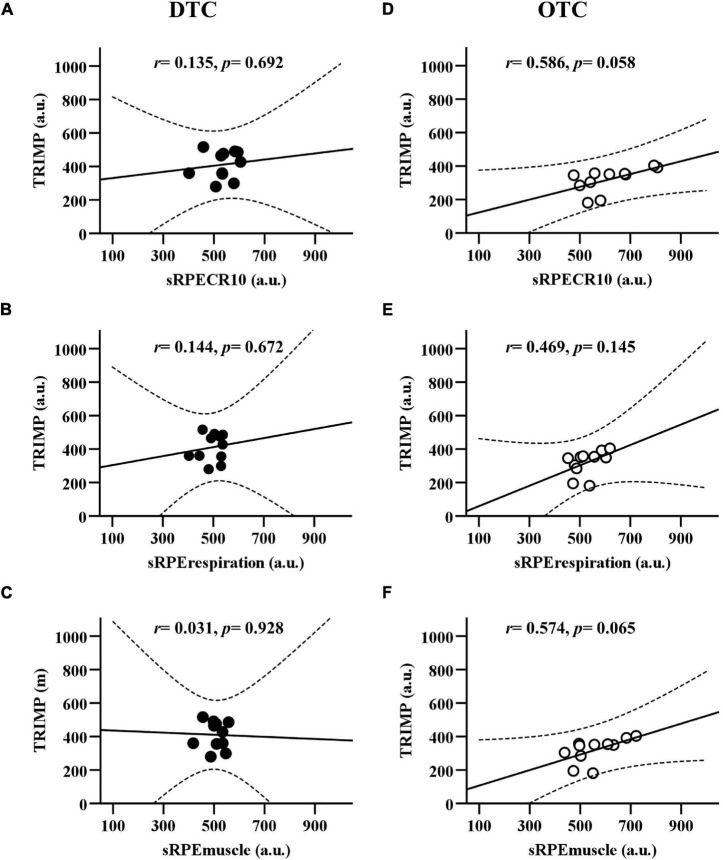
The linear relationship between session RPE (sRPE_CR10_, sRPE_respiration_, and sRPE_muscle_) and training impulse during domestic and overseas training camps. **(A)** Comparison between TRIMP and sRPE_CR10_ during DTC. **(B)** Comparison between TRIMP and sRPE_respiration_ during DTC. **(C)** Comparison between TRIMP and sRPE_muscle_ during DTC. **(D)** Comparison between TRIMP and RPE_CR10_ during OTC. **(E)** Comparison between TRIMP and sRPE_respiration_ during OTC. **(F)** Comparison between TRIMP and sRPE_muscle_ during OTC. TRIMP, training impulse; sRPE_CR10_, session rating of perceived exertion CR10; sRPE_respiration_, session respiratory perceived exertion; sRPE_muscle_, session muscular perceived exertion; DTC, domestic training camps; OTC, overseas training camps.

In Figure 5, the sRPE_CR10_, sRPE_respiration_, and sRPE_muscle_ demonstrated poor positive association to TRIMP during DTC (sRPE_CR10_: *r* = 0.135, *p* = 0.692; sRPE_respiration_: *r* = 0.144, *p* = 0.672; RPE_muscle_: *r* = 0.031, *p* = 0.928). But OTC (sRPE_CR10_: *r* = 0.586, *p* = 0.058; sRPE_respiration_: *r* = 0.469, *p* = 0.145; sRPE_muscle_: *r* = 0.574, *p* = 0.065) had higher positive association than DTC.

## Discussion

This study is the first to quantify training loads using RPE_respiration_ and RPE_muscle_ during DTC and OTC in male futsal players. The primary findings revealed that DTC demonstrated larger TD and TRIMP than that of OTC. Whereas RPE_CR10_ was significantly larger during OTC than that of DTC, no significant differences in RPE_respiration_ and RPE_muscle_ were observed between DTC and OTC. The second finding in this study was that RPE_respiration_ and RPE_muscle_ demonstrated a positive linear association to RPE_CR10_. Thus, either RPE_respiration_ or RPE_muscle_ can be used to quantify internal load during DTC and OTC in futsal players. Additionally, individual variability of measuring variables varied from camp to camp. Three different RPE scales showed similar dispersion and tendency of individual and group values across the DTC. Finally, TD and TRIMP are independent markers to training loads quantified by RPE scales during short-term futsal training camps.

We found that mean values of TD and TRIMP were significantly larger during DTC than that of OTC. It seems that there is a large demand of physical engagement during futsal DTC. Conversely, the RPE_respiration_ and RPE_muscle_ demonstrated no difference between the two different types of training camps. These findings highlighted the discrepancy of subjective and objective assessments of training loads in futsal DTC and OTC. Conversely, the different activity profiles that experienced between DTC (generally focused on fitness development, player selection, and technical evaluation) and OTC (usually incorporates more friendly matches and fewer training sessions to prepare for competition) could be a factor to explain these findings (Chen et al., 2020). Another possible explanation for these findings could be greater intersubject variability of RPE measures. Nevertheless, it is challenging to compare our observations with other populations, levels of players, or sports due to limited studies reporting internal or external loads during OTC.

It is interesting to note that the RPE_CR10_ was significantly larger during OTC than that of DTC. Although larger values of RPE_respiration_ and RPE_muscle_ were observed during OTC, the results did not approach statistical significance. Indeed, the RPE_CR10_ is a self-report tool to reflect the overall engagement of psychophyiolgoical efforts during the training or match. The higher values of RPE_CR10_ and sRPE_CR10_ reported during OTC might be related to a high frequency of friendly matches. Futsal is a high-intensity intermittent sports (Spyrou et al., 2020) with characteristics of quick decision-making on top of highly demanding technical and tactical performance (Corrêa et al., 2016). It seems that the players experienced higher perceptive loads rather than physiological strains during OTC as evidenced by TRIMP and TD metrics. However, the psychological stress and mental effort were not evaluated in this study. Thus, the overall contribution of psychological aspects on the intensity of RPE level is unknown.

We observed a positive association between RPE_respiration_ and RPE_muscle_ scales during both DTC and OTC. These findings are supported with previous studies that observed longitudinal changes in RPE_respiration_ and RPE_muscle_ scales in young professional adult soccer players during weekly training sessions and in-season matches (Los Arcos et al., 2014, 2016, 2017). The RPE_muscle_ and RPE_respiration_ scales showed similar features when quantifying the training load during weekly training sessions of a competitive period in professional soccer players (Los Arcos et al., 2017). Furthermore, different RPE scales permit researchers to obtain similar patterns of internal load during short-term specific training sessions. McLaren et al. (2020) recently demonstrated the usefulness of RPE_respiration_ and RPE_muscle_ scales in detecting improvements in high-intensity running profiles during a 2-week repeated sprint training intervention in semiprofessional soccer players. In this study, the positive association identified in all RPE scales indicates the potential usefulness of pictorial RPE measures in quantifying internal load in futsal training.

The results of linear regressions show no relationships between RPE scales and TD/TRIMP. This finding indicated independent markers of training load monitoring between the subjective and objective tools. Subjective assessment of training load can be used to understand the variation in an individual’s perception of daily changes in psychological and physiological status during sports training. Conversely, objective assessment of training load provides a quantitative measure of physiological responses and exercise performance to understand the accumulation of training and match loads. Collectively, our findings suggested no relationship between RPE scales and TRIMP/TD variables during futsal DTC and OTC. A combination of subjective and objective assessments to evaluate training loads in futsal training is recommended.

It should be noted that a within-subject approach was used during this study period (Costa et al., 2019, 2021; Figueiredo et al., 2021). Compared to TD and TRIMP, our results demonstrated that three different RPE scales showed similar dispersion and tendencies between individual and group values across the DTC. However, such observations may not exist during the OTC (see Figure 2). It is possibly related to the difference in training tasks among the OTC. In our study samples, the OTC consisted of several friendly matches to test the team’s competitive level. Costa et al. (2021) found that a training camp consisting of training sessions and friendly matches demonstrated a large variability of sRPE_CR10_ in female football players. Interestingly, there was a long interval between DTC 5 and DTC 6 (83 days) in our study. Despite the similar training contents with DTC 2, 3, and 4, a large dispersion of individual training load was observed in DTC 6. This finding was related to the initial fitness level before the training camp and competition status in the participants’ home teams (2-week break during the Chinese New Year, and no competition schedule). During the periodic training camps, coaches and strength and conditioning practitioners should consider the large individual difference in fitness level when the players return for training camps.

The first limitation of this study is that respiratory and muscular RPE used pictorial rating to quantify the perception of sensory feedback, compared to arbitrary units used in RPE_CR10_. This study players had extensive experience using the pictorial RPE_respiration_, RPE_muscle_, and sRPE_CR10_ measurements. Despite similar reports among different RPE scales found in our study, there still exists the potential that other populations, such as players with less training experience or those who are not familiar with this method, may show differing results. Second, the different perceived efforts might be related to the players’ initial fitness capacity. This study did not report the association between fitness capacity and individual perception of training stresses (Azcárate et al., 2020). A potential bias of the individual peak HR that changed across the timeline of training camps could be the third limitation of this study. The peak HR determined during the Yo-Yo intermittent recovery level 1 test in the first OTC may not be equivalent to actual HR_max_ in each training camp engagement. Finally, the task difference during DTC and OTC may result in large intraindividual variability in this study. The main focus of friendly matches during OTC could lead to a fluctuation of daily training loads and recovery status to more/less playing time per individual. Future studies need to measure these individual changes during the training camps.

The present findings revealed significant positive correlations among RPE_CR10_ and pictorial RPE_respiration_ and RPE_muscle_ during the futsal DTC and OTC. Our study reported that pictorial RPE measures and RPE_CR10_ have similar outcomes as monitoring tools during futsal training. Coaches and sports practitioners are encouraged to use one of the RPE measures concurrently with time spent in HR zones and TD measures during futsal training camps.

## Conclusion

In conclusion, TD and TRIMP of short-term DTC were larger than that of short-term OTC. However, the RPE_CR10_ is higher during OTC than that of DTC. No differences in perceived measures of muscular and respiratory RPE were identified. Additionally, three different RPE measures showed similar individual dispersion and group tendency across the DTC. The interpretation of perceived efforts *via* respiratory, muscular, and Borg CR10 quantifications provides a valuable resource in monitoring internal load in futsal DTC and OTC. No relationships among the perceived measures, TRIMP, and TD were observed in this study highlighting that it is essential to implement multiple tools when recording training loads in futsal players. Using a combination of subjective and objective measures to monitor training loads during short-term futsal training camps is warranted.

## Data Availability Statement

The raw data supporting the conclusions of this article will be made available by the authors, without undue reservation.

## Ethics Statement

The studies involving human participants were reviewed and approved by Human Ethics Committee of the University of Taipei. Written informed consent to participate in this study was provided by the participants’ legal guardian/next of kin.

## Author Contributions

Y-XL contributed to the study conceptualization, project administration, investigation, methodology, and writing (including reviewing and editing) of the manuscript. FMC and PB contributed to the study conceptualization and writing (including reviewing and editing) of the manuscript. ZC-M and C-HC contributed to the study data analysis and writing (including reviewing and editing) of the manuscript. S-CC contributed to the statistical analysis and writing (including reviewing and editing) of the manuscript. C-DK and Y-SC contributed to the study conceptualization, methodology, supervision, and writing (including reviewing and editing) of the manuscript. All authors contributed to the article and approved the submitted version.

## Conflict of Interest

C-DK is a consultant for Leadtek Research Inc. The remaining authors declare that the research was conducted in the absence of any commercial or financial relationships that could be construed as a potential conflict of interest.

## Publisher’s Note

All claims expressed in this article are solely those of the authors and do not necessarily represent those of their affiliated organizations, or those of the publisher, the editors and the reviewers. Any product that may be evaluated in this article, or claim that may be made by its manufacturer, is not guaranteed or endorsed by the publisher.
